# Nonexercise Activity Thermogenesis-Induced Energy Shortage Improves Postprandial Lipemia and Fat Oxidation

**DOI:** 10.3390/life10090166

**Published:** 2020-08-27

**Authors:** Chih-Hui Chiu, Che-Hsiu Chen, Min-Huan Wu, Yin-Fu Ding

**Affiliations:** 1 Graduate Program in Department of Exercise Health Science, National Taiwan University of Sport, Taichung 404, Taiwan; yingfuding@gmail.com; 2 Department of Sports Performance, National Taiwan University of Sport, Taichung 404, Taiwan; jakic1114@ntupes.edu.tw; 3 Sport Recreation and Health Management Degree Program, Tunghai University, Taichung 404, Taiwan; mhwu@thu.edu.tw

**Keywords:** energy expenditure, nonexercise activity thermogenesis, postprandial lipemia, physical activity, lifestyle, oral fat tolerance test

## Abstract

(1) Background: This study investigated the effect of nonexercise activity thermogenesis on postprandial triglyceride (TG) concentrations; (2) Methods: Ten healthy males completed a sedentary trial (ST) and a physical activity trial (PA) in a random order separated by at least 7 days. After each intervention on day 1, the participants consumed a high-fat test meal on the next day. The blood samples and gas sample were observed in the fasted state and for 4 h after consuming the oral fat tolerance test; (3) Results: The postprandial TG concentrations of total (AUC) (*p* = 0.008) and incremental area under the curve (IAUC) (*p* = 0.023) in the plasma of participants in the PA trial were significantly lower than those in the plasma of participants in the ST trial. The postprandial fat oxidation rate AUC of the PA trial was significantly higher than that of the ST trial (*p* = 0.009); (4) Conclusions: The results of this study indicated that nonexercise energy expenditure decrease the postprandial TG concentration and increase the fat oxidation the next day.

## 1. Introduction

Excessive increases in blood triglyceride (TG) levels after a high fat meal are associated with risk of cardiovascular diseases [1]. As a predictor, these excessive post-eating levels yield more accurate predictions than such levels during fasting [2,3]. The triglyceride, free fatty acids, and remnant cholesterol in the blood increase after high-fat meals. Increases in levels of these biochemical substances have been determined to be a major risk factor for atherosclerosis, myocardial infarction, coronary heart disease, and mortality [4]. The increased TG levels have been shown to be a better marker of cardiovascular disease independent of other lipids’ levels, nonfasting triglycerides and risk of myocardial infarction, ischemic heart disease, and death in men and women [5]. Because the high postprandial TG levels last for 6–8 h, and considering that we generally eat three meals per day, the postprandial lipemia level in our bodies is high most of the time. Thus, the decrease in postprandial lipemia (defined as high levels of triglycerides in the blood after a meal) is important for decreasing the likelihoods of insulin resistance and many types of cardiovascular diseases.

In a previous study, a single bout of endurance exercise was observed to reduce postprandial TG levels [6,7]. Exercise decreases postprandial TG levels 8 h after exercise, an effect that lasts for 48–60 h [8,9]. The mechanism underlying exercise’s reduction in postprandial lipemia levels potentially acts through increases in lipoprotein lipase activity (LPLA), insulin sensitivity, and fat oxidation rate [10]. Energy expenditure during exercise is a primary determinant of exercise’s effect on the postprandial lipemia reaction [11,12]. The greater such energy expenditure, the more noticeably exercise decreases postprandial lipemia levels. However, several studies have demonstrated that increases in the energy expenditure during exercise do not affect the postprandial lipemia reaction on the following day [10,13]. In our opinion, nonexercise activity thermogenesis (NEAT) explains this inconsistency in the present body of results.

Throughout the day, the human body expends energy through basal metabolism, as indicated by the basal metabolic rate (BMR), as well as through physical activities and the thermic effect of food. Physical activities can be divided into exercise and NEAT. Exercise constitutes only a small proportion of the energy expended through physical activity, with NEAT—comprising activities such as walking, performing heavy work, gardening, and fidgeting—constituting the majority [14,15]. Generally, NEAT is defined as comprising very low-intensity physical activities [14,15]. There are some studies that have examined the effect of NEAT on postprandial blood lipid levels. Miyashita (2013) demonstrated that sitting to rest and standing exerted similar effects on the subsequent day’s postprandial blood lipid reaction [16]. Crawford et al. (2020) and Kim et al. (2016) demonstrated that prolonged sitting increases the postprandial blood lipid levels compared to standing, regardless of energy balance or not [17,18]. As the exercise-induced energy shortage plays more important roles than diet on postprandial lipemia [19], it is important to know whether very low intensity exercise, defined as NEAT, induced the energy shortage that can decrease the postprandial TG concentration the next day. Therefore, to observe how physical activity affects the postprandial TG reaction, in the present study, the aim of this study was to investigate the effects of NEAT-induced energy shortage on postprandial TG concentration and postprandial fat oxidation after the consumption of high-fat meals in the subsequent day.

## 2. Results

### 2.1. Dietary Information on Day 1

Their meal contents included chicken sandwich and soy milk in breakfast; beef rice and seafood hand roll in lunch; Oyakodon and soy milk in dinner. The total energy consumed on day 1 was 2654.45 ± 274.7 kcal with 375.24 ± 43.11 g of carbohydrate (56.5 ± 1.53%), 79.78 ± 8.6 g of fat (27.07 ± 1.68%) and 113.83 ± 12 g of protein (17.63 ± 0.38%). The breakfast contained 785.6 ± 90.44 kcal, with 41.54 ± 1.95% energy from carbohydrate (81.2 ± 4.42 g), 38.29 ± 3.03% from fat (33.7 ± 7.27 g), and 19.25 ± 0.59% from protein (37.7 ± 2.84 g). The lunch provided 1138.7 ± 281.44 kcal, with 65.55 ± 1.65% energy from carbohydrate (186.14 ± 43.91 g), 21.44 ± 1.09% from fat (27.18 ± 6.91 g), and 13.43 ± 1.19% from protein (38.79 ± 12.39 g). The standard dinner offered 730.15 kcal, with 59% energy from carbohydrate (107.9 g), 23% from fat (18.9 g), and 22% from protein (40.5 g).

### 2.2. The Fasted State on the Morning of Day 2 of Each Main Trial

Table 1 shows the fasted state on the morning of day 2 of each main trial. The fasting insulin concentration of the PA trial was significantly lower than that of the ST trial (*p* = 0.019). The fasting fat oxidation of the PA trial was significantly higher than that of the ST trial (*p* = 0.029). There were no differences in TG, NEFA, glucose, glycerol and CHO oxidation.

### 2.3. The 24-h Energy Expenditure and Average Heart Rate

**Figure 1:**
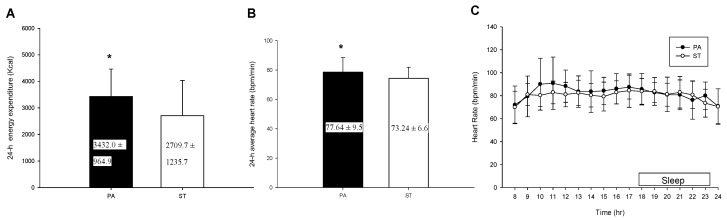
The 24-h energy expenditure (**A**), average heart rate (**B**) and 24-h heart rate (**C**). * mean a significantly different between the PA trial and the ST trial.

Figure 1A lists the two trials’ total energy expenditure over 24 h. The total energy expenditure over 24 h of the PA trial was significantly higher than that of the ST trial (*p* = 0.023). Figure 1B lists the two trials’ average heart rates over 24 h. The average heart rate over 24 h of the PA trial was significantly higher than that of the ST trial (*p* = 0.021), but there was no statistical difference in 24 h heart rate (*p* > 0.05) (Figure 1C).

### 2.4. Postprandial Fat Oxidation

**Figure 2:**
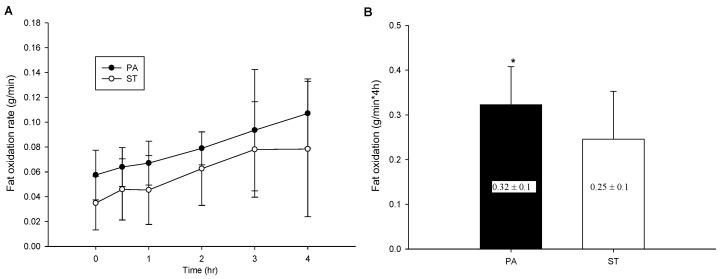
The postprandial fat oxidation over the 4 h (**A**) and fat oxidation area under the curve in 4 h (**B**). * mean a significantly different between the PA trial and the ST trial.

The postprandial fat oxidation rate of the two trials (Figure 2A) significantly differed but had no significant interaction (trial × time, *p* = 0.856; trial, *p* = 0.007; time, *p* = 0.001). As illustrated in Figure 2B, the fat oxidation rate area under the curve (AUC) of the PA trial was significantly higher than that of the ST trial (*p* = 0.009). The effect size (Cohen’s dz) was 0.59 for the fat oxidation AUC.

### 2.5. Postprandial TG Concentrations

**Figure 3:**
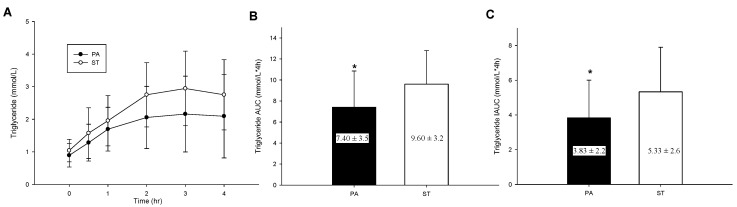
The postprandial TG concentration over the 4 h (**A**), TG total area under the curve in 4 h (**B**) and TG incremental area under the curve in 4 h (**C**). * mean a significantly different between the PA trial and the ST trial.

The postprandial TG level of the two trials (Figure 3A) significantly differed but had no significant interaction (trial × time, *p* = 0.052; trial, *p* = 0.009; time, *p* < 0.001). As illustrated in Figure 3B,C, TG total AUC (*p* = 0.008) and IAUC (*p* = 0.023) in the plasma of participants in the PA trial were significantly lower than those in the plasma of participants in the ST trial. The effect sizes (Cohen’s dz) were 0.67 for total AUC and 0.62 for IAUC.

### 2.6. Postprandial NEFA, Glycerol, Insulin and Glucose Concentrations

**Figure 4:**
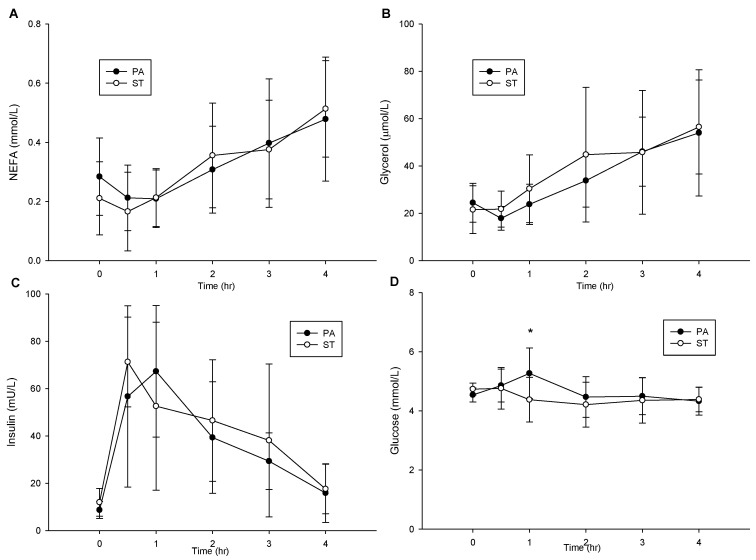
The postprandial NEFA concentration over the 4 h (**A**), the postprandial glycerol concentration over the 4 h (**B**), the postprandial insulin concentration over the 4 h (**C**), and the postprandial glucose concentration over the 4 h (**D**). NEFA, non-esterified fatty acids. * mean a significantly different between the PA trial and the ST trial.

There were no differences in postprandial NEFA (Figure 4A), glycerol (Figure 4B) and insulin (Figure 4C) (*p* > 0.05). The postprandial glucose concentration of the two trials (Figure 4D) had significant interaction (trial × time, *p* = 0.013; trial, *p* = 0.173; time, *p* = 0.057). The PA trial was significantly higher than the ST trial 1 h after a high fat meal (*p* = 0.015).

## 3. Discussion

This study’s results clearly indicated that, through lifestyle changes and previous-day NEAT (which consumes greater energy) prior to the consumption of a high-fat meal, next-day postprandial TG levels can be effectively decreased, and next-day postprandial fat oxidation rate can be effectively increased. In short, previous-day energy expenditure, prior to the consumption of a high-fat meal, is a major factor affecting next-day postprandial TG level and next-day fat oxidation rate.

To our knowledge, only three studies investigated the effect of nonexercise energy expenditure on next-day postprandial TG level [16,17,18]. Kim et al. (2016) examined the effect of increased NEAT with or without energy deficit (by increasing energy intake) and suggested that prolonged sitting increases the postprandial blood lipid levels compared to standing [17]. Crawford et al., (2020) asked the subjects to perform prolonged standing or sitting to induce a difference of 86 ± 29 kcal energy expenditure [18]. Energy replacement after exercise has been noted to affect the beneficial effect of exercise [20], therefore, it is important to know whether the very low intensity exercise, defined as NEAT, can induce the energy shortage that can decrease the postprandial TG concentration the next day. This study used nonexercise energy expenditure to examine the effect of different lifestyle patterns on next-day postprandial TG levels. This study’s results indicated that when the difference in energy expenditure, as monitored in heart rate between being sedentary and being active, is ~700 kcal, the next-day postprandial TG level was effectively decreased and the postprandial fat oxidation rate was effectively increased.

Previous studies using endurance exercises have demonstrated that a high level of energy expended through exercise greatly reduces next-day postprandial TG concentration [9,11]; the reason underlying this relationship, however, remains unclear. Evidence in the literature suggests that this relationship occurs through increases in LPLA, insulin sensitivity, and postprandial fat oxidation rate [9]. Although numerous studies have demonstrated that the amount of energy expended through exercise was instrumental to decreasing next-day TG level, other studies have noted that endurance exercise did not affect next-day postprandial TG level [10,13]. However, none of these studies tracked the energy expenditure in the 24 h before eating; their participants’ different lifestyle patterns after exercise potentially explain these studies’ nonsignificant research results. In our study, we tracked the participants’ energy expenditure through an indirect energy test, and participants were given strict instructions to adhere to their corresponding lifestyle pattern for 24 h. We noted that a sedentary lifestyle consumed ~700 kcal less than its active counterpart. Thus, an active lifestyle effectively decreased next-day postprandial TG level and increased next-day postprandial fat oxidation rate. Previous research has determined that walking consumed 200–300 kcal more than being sedentary, where this increased energy expenditure potentially decreased next-day postprandial TG level [21]. Therefore, the results of the present study indicate that with lifestyle changes toward greater engagement in NEAT, next-day postprandial TG level can be effectively decreased.

An increase in the fat oxidation rate might be one of the reasons why the TG level of this study’s PA trial was significantly lower than that of the ST trial. According to previous research using intermittent exercise, regardless of whether energy expenditure in such exercise is unchanged [10] or decreases [22] in comparison with endurance exercise, intermittent exercise contributes to a greater decrease in postprandial TG level. Excess post-exercise oxygen consumption (EPOC) might be the main reason why intermittent exercise is more effective than endurance exercise [23]. Increased EPOC through intermittent exercise can increase post-exercise energy expenditure. Thus, those who engaged in intermittent exercise have a significantly greater 24-h energy expenditure before eating relative to those who engaged in endurance exercise [24]. Conversely, previous studies have determined that the 24-h energy expenditure through intermittent exercise is similar to endurance exercise with lower exercise time [25]. However, no study has measured the energy expenditure after intermittent exercise. The results of this study indicated that increases in total energy expenditure, through a greater engagement in NEAT (~700 kcal), potentially increase the postprandial fat oxidation rate. Future research should track energy expenditure during the 24 h after intermittent exercise and observe changes in EPOC, the postprandial TG level, and fat oxidation rate.

Previous studies have noted that regardless of the duration and intensity of exercise, energy expenditure during exercise was the main factor underlying changes in LPLA levels and the postprandial TG reaction [9]. During exercise, only the muscle involved effectively alters LPLA in the muscle [26]. Greater levels of LPLA can decompose TG into NEFA and glycerol and decrease the postprandial TG concentration in plasma. Although several studies have discovered that LPLA levels in muscle can be increased through resistance training [27] or high-intensity exercise performed at 80% maximum oxygen consumption [28], these studies did not examine the effect of previous-day energy expenditure (before eating) on LPLA. Although, in this study, energy expenditure was increased through very low-intensity exercises—such as walking on streets, climbing stairs, doing housework, and watching stimulating movies—these low-intensity physical activities did not affect postprandial NEFA, glycerol and insulin concentrations, meaning there was no difference in postprandial endogenous fat metabolism. Nonetheless, the energy expenditure of these activities reduced the next-day TG level.

This study has two primary limitations. First, this research did not use Double Labeled Water and pedometers to examine the calorie burn and physical activity situation prior to the consumption of high-fat meals. In addition, since we did not use any pedometers or accelerometers to examine the physical activity, we cannot be sure if the ST group adhered completely to their guidelines, although they were not instructed to be as sedentary as possible. However, the indirect energy test used in this research has been used in other studies and has been proven to be effective [10,29,30]. Despite the possibility of error, the results clearly indicated a significantly greater 24-h energy expenditure in the PA trial than in the ST trial. Second, this research could not clearly differentiate between sources of calorie burn, whether from BMR, the thermic effect of food, or physical activity; we only identified the total calories burned. The day before consuming the high-fat meals, the strict instructions given to participants of each trial ensured uniformity in diet and lifestyle patterns within the trials. This measure enabled the inference that the calories burned through BMR and through the thermic effect of food were approximately equal. Furthermore, we also asked participants to abstain from exercise to ensure that most of their energy was expended through NEAT.

## 4. Materials and Methods

### 4.1. Participants

This study recruited 10 healthy adult men as participants (age 22.1 ± 0.3 years old, height 172.48 ± 5.8 cm, weight 72.83 ± 11.08 kg, body fat 22.5% ± 3.37%). The participants had not undergone physical training and had no habit of exercising. Participants were also free from hypertension, hyperlipidemia, heart disease, joint disease, osteoporosis, and other diseases due to which exercise is not recommended. Prior to the experiment, all participants were briefed about the risks involved in and the process of the experiment. Having agreed to participate, the participants read and signed consent forms, whose format was approved by the Institutional Review Board of the Tsoutun Psychiatric Center in Taiwan (No. 107007). The research team used a recruitment method, which yielded a similar sample size to their previous studies [10,31,32].

### 4.2. Experiment Design

The experiment had a crossover design, and the participants were randomly divided into a sedentary lifestyle trial (sedentary trial, abbreviated as ST) and a physical activity trial (physical activity trial, abbreviated as PA). On the morning of day 1 of the experiment, participants adopted their assigned lifestyle pattern. On the morning of day 2, they returned to the laboratory to eat a high-fat meal, and their postprandial blood TG reaction was observed. The experiments were conducted in a random order; to avoid interference from the first trial, the interval between each test was greater than 7 days.

### 4.3. Pre-Test

In this study’s pre-test, gas analyzers (Vmax Series 29C, Sensor Medics, Yorba Linda, CA, USA) were used to measure energy expenditure during rest and during lower-intensity exercise. After arriving at the laboratory, the participants wore heart rate monitors (Polar, Kempele, Finland) and gas analyzers. They laid face up to rest calmly for 20 min, and their heart rates and energy expenditure during rest were recorded. After their rest, to measure their energy expenditure during light physical activities, participants engaged in lower-intensity exercise tests. The participants first stood for 10 min on a treadmill at zero incline, and their energy expenditure during standing was measured using the gas analyzers. Subsequently, to understand the relationship between heart rate and energy expenditure through light physical activity, the participants underwent walking and jogging tests with gradual increments in speed (1, 2, 3, 4, and 5 miles per hour) at 3 min for each speed. The fat oxidation rates were calculated using the following Equations (1) and (2) [33]:

Fat oxidation (g/min) = 1.695 × VO_2_ − 1.701 × VCO_2_ (1)

Carbohydrate oxidation (g/min) = 4.585 × VCO_2_ − 3.226 × VO_2_ (2)

### 4.4. Experiment Procedure

The experiment occurred over two days. Three days prior to the first experiment, nutritionists gave dietary instructions to each of the participants. Specifically, participants were asked to abstain from alcohol and to consume fat, calories, and caffeine in moderation. Participants noted all the food they consumed during the three days prior to the experiment; they ate the same set of foods during the three days prior to the next experiment. Participants were also asked to avoid excessive physical activity, especially weight training and high-intensity exercise, during the three days prior to the experiment. On the morning of day 1 of the experiment, participants arrived at the laboratory between 8 a.m. and 9 a.m., rested and wore heart rate monitors to record their heart rate. They wore the monitors for 24 h until their consumption of high-fat meals the subsequent day. During the 24 h, participants’ meals were dictated by nutritionists. For both trials, the participants were provided food with calories that were 1.5 times their BMR, based on the mean of their energy expenditure [34]; the same meals were sent to their residences by delivery personnel in both trials. For the next 24 h, the participants were randomly assigned to ST or PA lifestyle patterns. ST participants were asked to be as sedentary as possible: they were encouraged to sleep, watch television, listen to music, or read at home. If they had to leave their home, they should, as much as possible, ride vehicles instead of walk. By contrast, PA participants were asked to engage in as much physical activity as they could for at least 6–8 h, other than exercise. Participants were encouraged to, for example, walk, climb stairs, picnic, and play stimulating video games. However, they were told to avoid purposeful exercise, such as hiking, jogging, and weight training. Other studies have used similar methods of recording energy expenditure and the same brand of heart rate monitors [10,30,31].

On the morning of day 2, all participants returned to the laboratory between 8 a.m. and 9 a.m. in a fasted state for a high-fat meal test. After the participants rested for 10 min, a peripheral venous catheter was inserted into their forearms, where their blood specimens were collected while they were in a fasted state. After the specimens were collected, the participants were served with regular high-fat meals. They then rested calmly in the laboratory for 4 h, where their blood lipid variation was observed. The procedure is detailed in Figure 1.

### 4.5. Oral Fat Tolerance Test (OFTT)

All meals for the OFTT that were planned by nutritionists and served to the participants had been used in previous research [10,31,32]. Their meal contents included toast, butter, cheese, cereals, and cream, providing 1.2 g of fat, 1.1 g of carbohydrates, 0.33 g of protein, and 16.5 kcal of energy, to each kilogram of body weight. The nutrition facts were determined according to the nutritional facts on the food packaging. During the experiment, the participants were asked to finish the meals for the OFTT in 15 min.

### 4.6. Blood Collection

During the experiment, blood was collected through the peripheral venous catheter (cannula Venflon 20G, BD Biosciences, Franklin Lakes, NJ, USA) and three-way connectors (Connecta Ltd., Stockholm, Sweden) from the veins in the arms (Antecubital fossa); 10 mL of blood was collected each time. Blood samples were collected 30 min before and after each meal, as well as at the first, second, third and fourth hours. Each time blood was collected, the catheters were washed clean with 10 mL of isotonic saline to ensure that blood did not congeal inside the catheters.

The collected blood was immediately placed into blood collection tubes containing EDTA. The hematocrit of each blood sample was analyzed using a cell counter analyzer (Sysmax KX-21N, Sysmex, Kobe, Japan). Subsequently, the blood samples were centrifuged at 500× *g* and at 4 °C for 20 min. Plasma specimens were then obtained and immediately stored in a refrigerator at −80 °C until the biochemical analysis.

### 4.7. Blood Biochemical Analysis

The TG, non-esterified fatty acids (NEFA), glucose, and glycerol (7020, Hitachi Koki, Tokyo, Japan) levels in plasma were analyzed using a commercial reagent (through the GOD-PAP method, Randox, Crumlin, UK). The insulin levels in plasma were analyzed using a chemiluminescence analyzer (Elecsys 2010, Roche Diagnostics, Basel, Switzerland) combined with a commercial reagent (Roche Diagnostics, Basel, Switzerland). The inter-assay and intra-assay CVs were: TG (1.9% and 0.6%, respectively); NEFA (2.6% and 4.4%, respectively); glucose (2.2% and 3.7%, respectively); glycerol (0.9% and 6.4%, respectively); insulin (0.8% and 2.6%, respectively).

### 4.8. Statistical Analysis

All data are presented in terms of their average ± standard deviation. The Shapiro–Wilk test was used to first assess the normality of data. The trapezoidal rules were used to calculate for blood parameters versus time curve (AUC). The blood biochemical data for each period and trial were analyzed using two-way ANOVA with repeated measures. The Bonferroni method was used for a post hoc comparison if the difference was statistically significant. The fasting serum biochemistry, 24-h energy expenditure, average heart rate, fat oxidation area under the curve (AUC) and TG AUC were analyzed using paired samples t-test. A sufficient sample size of 8 participants was calculated using G*power 3 with an alpha value of 5% and a power of 0.8. The significance level was set at α < 0.05.

## 5. Conclusions

The results of this study indicated that increased nonexercise-induced energy expenditure decreased the postprandial TG concentration and increased the fat oxidation the next day. The results indicated that activity-based lifestyles effectively improve the TG reaction and fat oxidation rate after the consumption of a high-fat meals, thus providing another effective mechanism for improving health outcomes.

## Figures and Tables

**Table 1:** The participants’ physiological information and fasting serum biochemistry.

	PA	ST	*p* Value
TG (mmol/L)	0.89 ± 0.36	1.04 ± 0.34	0.065
NEFA (mmol/L)	0.28 ± 0.13	0.21 ± 0.12	0.173
Glucose (mmol/L)	4.60 ± 0.3	4.76 ± 0.2	0.116
Insulin (mU/L)	8.98 ± 3.6	11.8 ± 5.6	0.019
Glycerol (μmol/L)	24.4 ± 8.2	21.5 ± 10.1	0.458
fat oxidation (g/min)	0.06 ± 0.02	0.03 ± 0.02	0.029
CHO oxidation (g/min)	0.16 ± 0.04	0.19 ± 0.07	0.212

Values are mean SD, n = 10. PA, physical activity trial; ST, sedentary trial; TG, triglyceride; NEFA, non-esterified fatty acids.
